# Emerging Technologies for Aerial Decontamination of Food Storage Environments to Eliminate Microbial Cross-Contamination

**DOI:** 10.3390/foods9121779

**Published:** 2020-11-30

**Authors:** Márcia Oliveira, Brijesh K. Tiwari, Geraldine Duffy

**Affiliations:** 1 Food Chemistry and Technology Department, Teagasc Food Research Centre, Ashtown, D15 KN3K Dublin, Ireland; marcia.oliveira@teagasc.ie; 2 Food Safety Department, Teagasc Food Research Centre, Ashtown, D15 KN3K Dublin, Ireland; geraldine.duffy@teagasc.ie

**Keywords:** aerial microbial contamination, cold storage room, decontamination technologies, food industry

## Abstract

Air is recognized as an important source of microbial contamination in food production facilities and has the potential to contaminate the food product causing food safety and spoilage issues for the food industry. Potential for aerial microbial contamination of food can be a particular issue during storage in cold rooms when the food is not packaged and is exposed to contaminated air over a prolonged period. Thus, there are potential benefits for the food industry for an aerial decontamination in cold storage facilities. In this paper, aerial decontamination approaches are reviewed and challenges encountered for their applications are discussed. It is considered that current systems may not be completely effective and environmentally friendly, therefore, it is of great significance to consider the development of nonresidual and verified decontamination technologies for the food industry and, in particular, for the cold storage rooms.

## 1. Introduction

Air may contain suspended microdroplets of liquid, solids and living substances, such as microorganisms. A range of biological agents including plant cells, dust, parasites, bacteria, yeasts and moulds and viruses can be found in the air [1]. Different pathways, which can release microorganisms into the aerial environment, include wind, air flow, rain and water splashing, organic matter and dust release from surfaces.

Several studies have reported airborne microbial transfer in food production environments. For example, airborne bacteria presented an increase in 25- to 30-fold after rain in production fields [2,3]. Additionally, a study showed the possibility for *Salmonella* to be carried through the rain and air and further contaminate tomatoes at high levels [4]. These pathogenic bacteria have been found in the air of poultry [5] and pig production environments [6], and that has also been stated as a significant pathogen for rapid transmission through the air in turkey [7] and pigs [8].

Air is well recognized as a source of microbial contamination in food production facilities. The aerial microbial load and diversity will be impacted by the microbial contamination on the raw intake materials, the air-flow within the plant, movement of people, and the length of time the product is exposed to air. There may also be direct interaction of the product with the air, if air is used for cooling or fluming of the product. The microbiota present in the air has the potential to cause foodborne disease and reduce the shelf-life of the food products resulting in potential food safety issues and economic loses for the food industry [3,6,8].

This review aims to highlight the importance of air as a source for microbial contamination in chilled in door environments in the food industry and to provide an overview of current applications for aerial decontamination in food chilling rooms. The potential challenges of the approaches and technologies that can be applied are discussed.

## 2. Sources of Airborne Contamination in Processing Plants

Nowadays, the monitoring of the air for microbial contamination in the food industry has been accepted as an important standard quality control, therefore, food producers are including it as part of their Hazard Analysis Critical Control Point (HACCP) system [1]. This fact shows the great importance of airborne route as a potential cause of microbial contamination. The microbiota in the air can vary significantly in both composition and concentration between food factories, in different areas within a food plant depending on the activity carried out there, i.e., clean/dirty areas, being bacteria most frequently found than other types of microorganisms in the food-processing air [9].

The survival of airborne microorganisms indoors is significantly influenced by several environmental factors. These factors include temperature, relative humidity (RH), atmospheric gases, ultraviolet irradiation and surrounding organic material [10,11]. Air temperature and RH are the factors that most affect the persistence and spread of airborne microorganisms indoors [12]. Some pathogenic bacteria such as *Salmonella* [6,13], *Escherichia coli* [14] and *Listeria* [15] are able to survive in the air and have the potential to contaminate food products due to their high tolerance to environmental factors.

**Figure 1:**
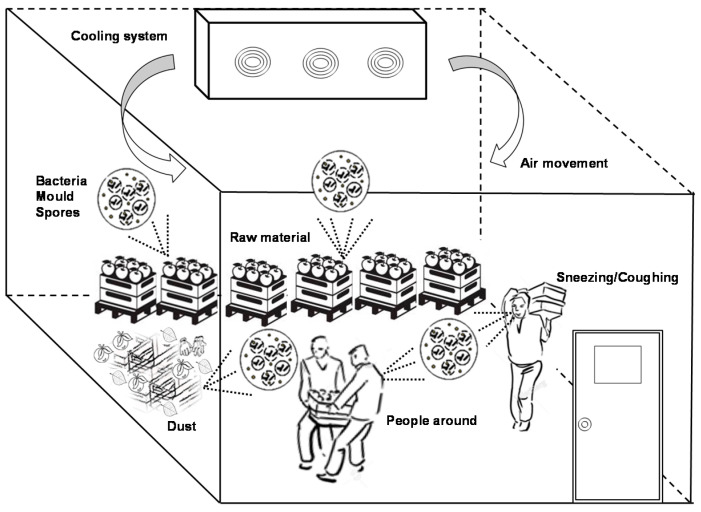
Schematic design of air contamination sources in a fruit cold storage room.

The presence and concentration of bacteria, yeasts and mould spores in food facilities may be due to: raw materials, movement of equipment, small distance between areas, open drains, high levels of moisture, plant layout, people movement in an area, poor hygiene and dust (Figure 1) [9].

Several sources of airborne contamination inside food facilities can be reported. In two herb-processing factories was found high counts of airborne microorganisms and endotoxins [16]. The levels of airborne microorganisms were 40.6–627.4 × 10^3^ cfu/m^3^. In a fresh produce facility, the water splashing during the salad washing was shown to be a potential source of aerosols from the water droplets containing microorganisms [17]. Buckland and Tyrell [18] observed that aerosols from nasal secretions (sneezing and blowing) were much higher than from coughing.

From a food processor perspective, it is essential to know if the contaminated air is a potential source of contamination for the food product. Burfoot et al. [19] observed that the airborne bacteria from evisceration room did not increase the high levels of bacteria on the poultry carcasses. In another study, Burfoot et al. [20] reported that surface contacts were more important for carcasses contamination than airborne bacteria in cattle and lamb slaughterhouses. However, other studies highlighted the importance of airborne contamination on beef carcasses [21,22].

Therefore, air can be recognized as a vector for microbial contamination in a range of food processing plants, including dairy [23], fruits [24], pork [6], poultry [19] and beef [20].

### Cold Room Microbial Cross-Contamination

As detailed above, microbial air contamination can occur at various points during food processing and via different transmission routes. However, there is little research on the air quality in food chill environments. It is documented that there can be a significant level of aerial microorganisms, providing a source of food contamination, which could negate the impact of any prior intervention step.

Okraszska-Lasica et al. [25] examined the levels of bioaerosols in Irish beef and sheep factories and showed mean total viable counts (TVC) of 1.75–2.34 log cfu/m^3^ in the chill room, highlighting the potential of air as a source of carcass contamination during chilling. Authors observed high levels of airborne TVC from a chilling (3.28 log cfu/m^3^) and a spray-chilling (4.16 log cfu/m^3^) rooms, in poultry slaughtering and processing plants. Enterobacteriaceae counts were found at 2.02 log cfu/m^3^ in the air-chilling and 2.06 log cfu/m^3^ in the spray-chilling room [26]. In another poultry abattoir, airborne bacteria in chilling facilities were also examined [27], and the authors reported that the microbial load in the air of two poultry plants was quite different, consisting mainly of Micrococcaceae and Gram-positive irregular rods.

Regarding fresh produce storage facilities, postharvest pathogenic spores originated from infected fruit and vegetables can be considered the source of air contamination in packinghouses [28]. Penicillium, Alternaria, Cladosporium, Fusarium and Botrytis are the most severe postharvest diseases and cause serious losses during storage period.

Recognising that indoor air can be a vehicle for food contamination, this review will look for current applications of aerial decontamination in the chill room and potential challenging approaches that can be applied. It is considered that current systems may not be completely effective and environmentally friendly, therefore, it is of great significance to consider the development of nonresidual and verified decontamination technologies for the food industry and, in particular, for the cold storage rooms.

## 3. Current and Emerging Air Decontamination Methods

An appropriate level of hygiene in food production facilities is required by means of an effective cleaning and sanitation programs. In the case of food facilities, a complete sterile environment is unrealistic and unnecessary, therefore, to achieve good levels of hygiene, the sanitation process is the most suitable, i.e., eliminate pathogenic microorganisms and high number of spoilage microorganisms [29].

As mentioned previously, the clear association of airborne and food contamination has led to a corresponding rise of products and technologies that declares for safe and effective decontamination of air [30]. Several processes and technologies have been described for their application on environmental surface decontamination; however, some limitation can be observed on the number and variety of methods for indoor air decontamination. However, some studies have been carried out on approaches and technologies to reduce the air contamination in food cold storage rooms (Table 1).

Chemical disinfectants are routinely used for the control of bacteria and mould spores in food production facilities. With an increase in consumer awareness and the demand for environmentally friendly technologies, there is a need for alternative methods to control both the air and exposed surfaces in a processing area, an approach that can achieve the entire room decontamination. Additionally, using liquid sanitizers makes it difficult to achieve a complete and exhaustive cleaning and decontamination of a cold room. Such approaches have been used to decontaminate entire areas in the pharmaceutical and clinical sectors, but very less information exists on the application of these techniques in food processing facilities [41].

The number of techniques designed for complete room decontamination is increasing, but those that are commercially available include chemical fogging, hydrogen peroxide vapour, ozone, chlorine dioxide, ultraviolet light and ionisation.

### 3.1. Ultraviolet Light (UV) Irradiation

UV light has the capacity to break the molecular bonds in DNA, and thereby can inactivate the microorganisms. Shortwave UV radiation (UVC, 254 nm) has been shown to reduce the microbial load both in air and on hard surfaces free of organic residues [42]. The efficacy of UV irradiation is a function of many different parameters such as intensity, exposure time, lamp placement, and air movement patterns.

The disinfecting capacity of UV is well known and widely used in medical and veterinary practices as well as in decontamination of air, surfaces and instruments [43,44]. Burfoot [45] reported the use of a UV system that could reduce microbial populations at rates over 99% in air flows up to 2 m^3^/s. Microbial air quality in cold stores and egg-hatching cabinets has also been improved using UV radiation units [42]. The UV radiation has been shown to be able to reduce the airborne microbial counts by 4 log units [46,47]. Bodmer [48] showed that UVC radiation was beneficial in decontamination of air in meat processing plants; the treatment destroyed bacteria, yeasts and moulds, as well as viruses.

UV treatment has some advantages over chemical sanitizers such as no chemicals are added to or remain in the product, instantaneous and specific biocidal action, easy to install and relatively maintenance-free, low capital costs and no chemical storage hazards [49].

#### Application of UV in Cold Storage Room

**Figure 2:**
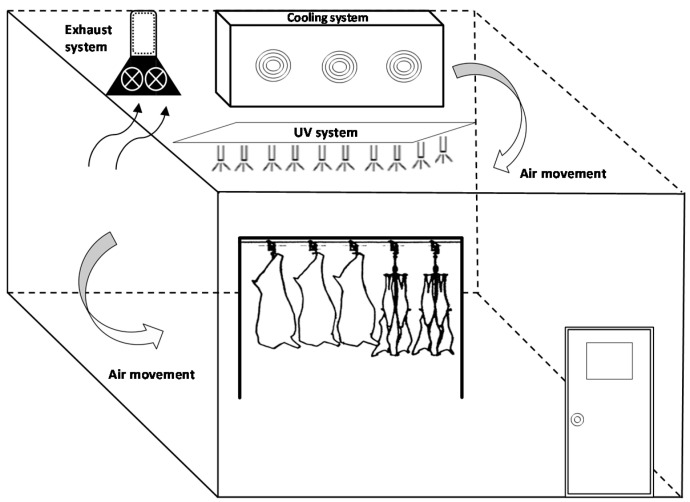
Schematic design of UV treatment in a meat cold storage room.

The application of UV for decontamination of indoor air is well known; however, to the best of our knowledge, little information exists on their effectivity in cold storage rooms in food environments. Physical decontamination techniques for the cold storage facility are favourable compared to wet delivery of chemicals. It is plausible to assume that UV technology is one of the most attractive options to achieve this objective. A schematic example of UV light installed in a meat cold storage room is shown in Figure 2.

A study carried out by Saks et al. [31] reported the efficacy of Pulsed UV light (PUV) in cold storage rooms to eliminate postharvest pathogens in comparison with ultrasonic fogging with hydrogen peroxide. The cold storage rooms used in this study was 11 m^3^ sizes and the PUV treatment was applied for 66 or 1000 s. Air sampling without entering the room after the PUV application showed very low microbial air counts, especially 24 h after the treatment. On the other hand, entering the rooms apparently increased the microbial air counts after the PUV application. The application time of 66 s yielded similar results as 1000 s, suggesting that short exposure times may be sufficient for eradication of microbial contamination. The authors suggested an up scaling and optimization of the treatment in order to realize its potential under commercial conditions.

A combination of technologies including filtration, electrostatic polarization and UV light was incorporated in one unique system (console unit) and was demonstrated to reduce airborne microorganisms in a meat processing plant [32]. The decontamination study was done in a carcass chill storage room and in the aging cooler. The cold room (volume of 128.3 m^3^) at temperature between 0 and 2 °C and maximum airflow of 21.3 m/min was used for chilling the carcasses after slaughtering, while the aging cooler (volume of 364.1 m^3^) at the same temperature and maximum airflow of 7.9 m/min was used for the storage and aging of carcasses and other meat products. This UV system used a germicidal bulb (100 mW/cm^2^) with an airflow of 10.61 m^3^/min. In this study, the console unit combining three technologies was effective to reduce 1.0–1.5 log units of airborne bacteria and moulds in the aging and chill rooms. The authors highlighted the effectiveness of this console unit to decontaminate airborne microorganisms in a small processing plant.

### 3.2. Oxygen-Based Technologies

#### 3.2.1. Fogging

**Figure 3:**
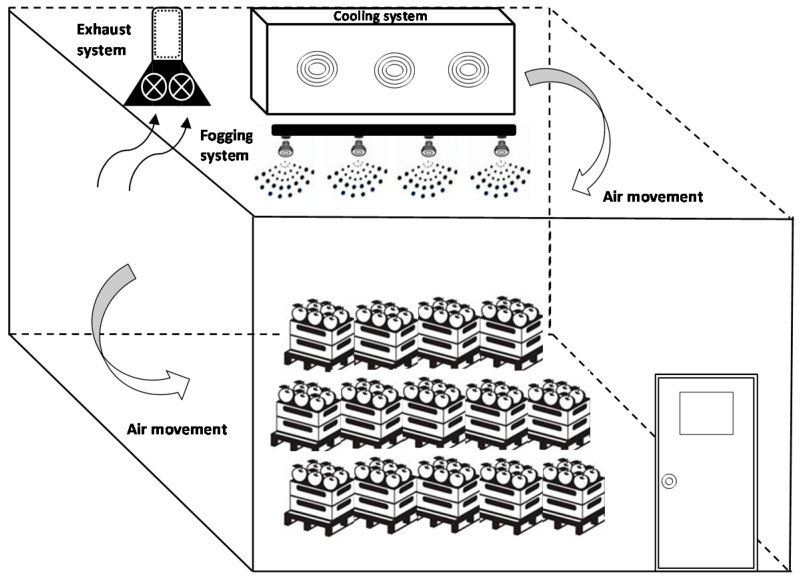
Schematic design of a fruit cold storage room with a fogging treatment.

Various oxidizing agents with a demonstrated antimicrobial activity have been considered as suitable contact surface sanitizers including chlorine dioxide (ClO_2_), organic acids, hydrogen peroxide (H_2_O_2_) and ethanol [50,51]. The use of gaseous sanitizers present advantages over the liquid disinfectants by being more spreadable, uniform and reachable to difficult/hidden areas more easily improving their antimicrobial capacity [51,52,53]. Therefore, the purpose of fogging is to enhance the application of such sanitizers and to reduce the number of airborne microorganisms. Fogging implies dispersal of finely disposed droplets (Figure 3) of a disinfectant and is applied after cleaning to guarantee that all surfaces and equipment in the food processing plant have received the sanitizer.

Most of the research using fogging as a sanitizer has been done for medical and pharmaceutical proposes with contradictory results, while little research has been carried out regarding food processing plants and equipment.

Hedrick [54] found that a chlorine fog can reduce the airborne counts. However, Holah et al. [46] found that fogging was less effective as compared to other decontamination methods such as the use of ozone or ultraviolet radiation. Burfoot et al. [55] showed that fogging was effective to reduce bacterial counts on upward-facing surfaces but not on vertical or downward-facing surfaces. They also concluded that fogging reduced airborne microorganisms’ levels and the fogging drop size and dispersion are important factors for this procedure to be effective.

There are some concerns regarding the application of fogging mainly related to the high concentrations of the disinfectants that can adversely affect workers’ health. These issues need to be taken in account before their commercial implementation [50,55].

There are many different sanitizers used as the fogging solution but hydrogen peroxide and chlorine dioxide are the most commonly preferred.

Hydrogen peroxide is a highly active biocide that exhibits activity through the generation of hydroxyl free radicals that penetrate the cell wall to attack lipids, proteins and DNA [56]. It has a wide range of activity on vegetative cells of bacteria as well as on spores (bacterial and fungi) and viruses but less activity against catalase-positive microorganisms.

The application of hydrogen peroxide produces no residues since it decomposes to water and oxygen. It can be used as liquid or in vapour phase, frequently, in combination with heat, since high temperature increases its antimicrobial ability [57]. Hydrogen peroxide vapour technology has been used primarily in the medical, biological and pharmaceutical industries, but is commercially available and has demonstrated capacity to decontaminate large spaces.

This sanitizer has been suggested as a possible alternative to formaldehyde within degreening rooms in citrus packinghouses to reduce postharvest pathogens [58]. Additionally, hydrogen peroxide fogging was applied to reduce human pathogens in contaminated environments and on surfaces [59].

Chlorine dioxide is a strong oxidizing agent and was developed for water disinfection applications; its disinfection activity is known to be less influenced by pH and results in less harmful by-products than traditional chlorine treatment [60]. Chlorine dioxide gas has equal or greater antimicrobial potency than chlorine. Its antimicrobial activity is primarily due to its destabilizing effects on cell membranes and also oxidizes cell-surface proteins [61]. Due to its high oxidative capacity, the required amount of chlorine dioxide is lower and the required contact time is shorter to obtain the same antimicrobial effect as chlorine [62]. Regardless of its practical applications, it can be toxic to humans at concentrations greater than 1000 ppm [63]. Furthermore, chlorine dioxide gas has to be generated on-site, because it cannot be compressed and stored or transported under pressure [64]. This may have some practical limitations for the potential applications due to the storage and transportation.

Chlorine dioxide has been effectively used for environmental decontamination, particularly within food processing environments [64]. Interestingly, although gaseous or liquid systems are widely used, there is little information on the delivery of this sanitizer as a biocide for air decontamination.

Jeng and Woodworth [65] reported the use of chlorine dioxide vapours as disinfectant in an attempt to find a substitute of ethylene dioxide gas for medical products. They proved that it can inactivate spores of *Bacillus* species. In addition, this disinfectant has been fumigated in buildings to control several fungi like *Stachybotrys chartarum*, *Penicillium chrysogenum* and *Cladosporium cladosporioides* [66].

##### Application of Fogging in Cold Storage Room

As mentioned above, studies on hydrogen peroxide vapour and chlorine dioxide gas decontamination of cold food storage areas are limited. Saks et al. [31] compared the efficacy of PUV (discussed before in this article) and ultrasonic fogging with stabilized hydrogen peroxide for decontamination of air and surfaces of cold storage facilities. They found both treatments were effective. Stabilized hydrogen peroxide reduced microbial counts in the air on the day of treatment; however, an increase in air counts was observed 24 h later.

Sholberg [33] reported, using an unpublished data by W. McPhee, that StorOx^®^ applied by cold fogging at dilution rates of 1:50 or 1:30 in one and 12 rooms, respectively, reduced mould contamination to low levels compared to the untreated control. StorOx^®^ is a broad-spectrum sanitizer, containing a mixture of hydrogen peroxide and peroxyacetic acid and was tested in several pome fruit cold storage rooms in the Pacific North West.

On a strawberry cold storage room, Vardar et al. [34] evaluated the efficacy of several sanitizers such as hydrogen peroxide, chlorine dioxide, citric acid, sodium hypochlorite and ethanol applied by fogging to reduce postharvest pathogens. All sanitizers were effective to reduce airborne microorganisms in the cold storage room. However, differences can be found between concentrations of each treatment applied. Similarly, Karabulut et al. [35] observed that chlorine dioxide applied by fogging reduced microbial counts in storage rooms of figs.

Daus et al. [67] simulated a storage facility environment and studied the application of ultrasonic fogging to control postharvest pathogens. In this study, the efficacy of ultrasonic fogging and direct application of didecyldimethylammonium chloride (Sporekill^®^) were compared. The direct exposure of the sanitizer was more effective to reduce *Botrytis cinerea* conidia and the ultrasonic fogging treatment requires a higher concentration to achieve the same efficacy.

The air of the packinghouse may carry an abundance of pathogenic spores, which may originate from infected fruit and vegetables, or from plant remains in the packinghouse or its surroundings [28]. Therefore, there is a fundamental need for effective sanitation treatments in packinghouse environments and the application of fogging treatments can be used for the control of postharvest microorganisms in such facilities.

#### 3.2.2. Ozone

There are several ways to produce ozone and many applications by which to apply it. In the case of food processing applications, ozone is generated commercially within the food industry by one of two generally accepted procedures: by passing an oxygen-containing gas through either a source of UV radiation or a high-energy electrical field. These methods are known as photochemical (UV) and corona discharge (CD), also called plasma technique [68].

Ozone has been widely used as a disinfectant due to its oxidising power. Its most common application is in the disinfection of water; however, it is being tested mainly for the disinfection of air. Ozone may well be the most potent biocide among all, more than chlorine, chlorine dioxide, hydrogen peroxide or peracetic acid [69]. The broad-spectrum application may make ozone a greener alternative to traditional approaches for various food processing applications. Ozone can be used in a gaseous or in aqueous phase and decomposes rapidly in air producing oxygen, and thus generally leaves no residues. Ozone applications in food processing have been legally approved, although to varying degrees, in North America, Australia, New Zealand, Japan and several European countries [70].

Ozone is a strong antimicrobial agent with a wide range of activity and its efficacy has been documented against bacteria, fungi, spores, protozoa and viruses [71,72,73]. Its effectiveness to kill microorganisms is a function of concentration, temperature, length of exposure and the substrate on which they reside as well as of the target microorganism.

Kim and Yousef [74] tested ozone against *Pseudomonas fluorescens*, *E. coli* O157:H7, *Leuconostoc mesenteroides* and *L. monocytogenes*. Exposure to 2.5 ppm for 40 s produced 5.0–6.0 log decrease in numbers, with *E. coli* O157:H7 being the most resistant. A report by Taylor and Chana [75] indicated a 2.0 log reduction in both airborne and surface-adhered *Pseudomonas aeruginosa* in 2 h when exposed to 2 ppm ozone. In the ozonation experiments carried out in an aerobiology cabinet [46] exposure of 4 µg/mL for 5–10 min reduced the number of airborne *P. aeruginosa* significantly with 2–4 log units. In a cheese storage room at temperature of 2–4 °C and 85–90% RH, Gabriel’yants’ et al. [76] treated it with 2.5–3.5 ppm of ozone for 4 h at 2- to 3-day intervals. The results showed that mould growth was prevented in stored Russian- and Swiss cheeses and packaging materials for more than 4 months with no negative effects on the sensory properties and chemical composition of the cheese. Additionally, Cullen and Norton [77] reported that ozonation is an efficient technology for inactivation of airborne moulds.

##### Application of Ozone in Cold Storage Room

Although some authors reported the efficacy of ozone to decontaminate airborne microorganisms, the results found in the literature are limited to the application in cheese ripening and storage rooms to prevent airborne moulds. Ozone has been also used in packinghouse storage facilities but is more commonly associated with surface and fresh produce decontamination than the indoor air. Some systems rely on the continuous presence of ozone in storage room air but to the best of our knowledge, no data exist about the airborne microorganisms monitoring.

One of the first works using ozone in cheese-storage facilities was published by Gibson et al. [36]. They studied the application of different ozone concentrations to control a well-established mould growth and reduce its progress on Cheddar cheese. The high-ozone (3–10 ppm) and low-ozone (0.2–0.3 ppm) treatments reduced the mean mould spore counts in the curing rooms by 94% and 88%, respectively. Some years later, Shiler et al. [37] reported that ozone concentrations of approximately 0.05 and 5 ppm in the air of a cheese ripening room inactivated 80–90% and 99% of mould spores, respectively.

Subsequently, a cheese ripening room was ozonated for 20 weeks, and the effectiveness of the treatment was monitored both in the air and on the surfaces of the room [38]. The cheese ripening room (3500 m^3^) with a constant airflow rate injection was kept at 5 ± 1 °C and 80% RH with an ozone production rates of mainly 8 g/h. The ozone treatment used in this study was very effective to reduce mould spores in the air of the cheese ripening room.

In a more recent study, Pinto et al. [39] assessed the efficiency of gaseous ozone fumigation in a maturation room of parmesan cheese type Grana to control fungi suspended in the air. The cheese ripening room with 250 m^3^ was treated with 4 ozone generators (30 mg O_3_/h) that produced 0.48 mg O_3_/m^3^. After a 40 days treatment, a decrease in 2.07 log cycles in fungal viable counts in the atmosphere was observed. Similarly, Lanita and Silva [40] studied the application of ozone during 60 days in a Parmesan-type cheese ripening room and found a 63% reduction in airborne yeasts and moulds.

It is well described that the environment in the cheese ripening rooms facilitates the mould growth; consequently, the stored cheeses will most likely become contaminated. Based on those results, ozone treatment could be a promising and effective technology to control airborne microorganisms in cold storage rooms.

### 3.3. Other Decontamination Techniques

A photocatalysis using titanium dioxide (TiO_2_) is an advanced oxidation process and has been pondered as a possible alternative to conventional physical decontamination processes [78,79,80]. Goswami et al. [81] reported for the first time the bactericidal activity of TiO_2_ photocatalysts in indoor air. Since then, numerous studies related to the bactericidal effect and photomineralization of the TiO_2_ photocatalyst have been conducted to the inactivation of bacteria and viruses [82,83,84,85]. Ye et al. [86] developed a new technique for an air cleaner in cold storage environments for reducing the occurrence of postharvest moulds enhancing the efficiency of disinfection of the photocatalytic process. For that, they studied the photocatalytic sensitivity of *P. expansum* spores to an activated carbon fibre (ACF)-supported TiO_2_ photocatalyst (TiO_2_/ACF) or a silver-doped TiO_2_/ACF photocatalyst. It was compared the performance of photocatalysis (PC) on silver-doped TiO_2_/ACF photocatalyst prepared by an ion sputtering method using two different techniques and the performance of photoelectrocatalysis (PEC) on TiO_2_/ACF film for the enhanced disinfection. Both techniques applied in this study improved the inactivation of the airborne fungus with an optimized light intensity of 2.3 mW/cm^2^ and a bias voltage of 66.7 V.

Recently, Skowron et al. [87] studied the application of the Radiant Catalytic Ionization (RCI) cell to generate oxidative gases, such as ozone and peroxide, to reduce several airborne bacteria and moulds. This technique uses UV radiation and appropriate photocatalysts, such as TiO_2_, an appropriate wavelength and a photooxidation process, which integrate the hydrophilic coating of surface of matrixes in the RCI module [88]. In this study, airborne *E. coli* and *Candida albicans* were completely inactivated. Additionally, *S. aureus*, *S. epidermidis* and *Enterococcus faecalis* populations were reduced at 99.9%. Lower effectiveness of RCI was recorded in the case of spore-forming bacteria (from 96.6% to 98.9%) and moulds (from 96.8% to 99.4%). *Clostridium sporogenes* spores were less sensitive to RCI with 71.7% of reduction in the air [87].

There is still an increased interest in technologies using exposure to light for inactivation of microorganisms. For example, photodynamic inactivation (PDI) is a promising treatment using visible-light inactivation that needs the incorporation of photosensitizing molecules [89,90,91,92]. Maclean et al. [93] investigated the use of light-emitting diodes (LEDs) with a specific wavelength of 405 nm. This work was performed in order to reduce methicillin-resistant *Staphylococcus aureus* (MRSA) and other important human pathogens (both Gram-positive and Gram-negative bacteria). Both Gram-positive and Gram-negative bacteria were effectively reduced and regarding MRSA, this technique achieved a complete elimination of this microorganism even at high concentrations. The authors concluded that this technology needs further development; however, is a promising technique to provide effective air decontamination with a specific wavelength of 405 nm visible-light from LED source and can be applied in both clinical and nonclinical facilities.

Another prominent technology is electrolyzed oxidizing water (EOW) that has been recognized as a feasible biocide [94,95]. Their principles and applications on food processing is not completely new. In general, the alkaline and acid water are produced by passing a diluted noniodized salt (NaCl) through an electrolytic cell, where the pH of the generated alkaline water is about 11–12 while the pH of acid water is around 1–3. It is considered that the alkaline solution (NaOH) has a cleaning effect, whereas the acid solution (HOCl) has a strong biocidal activity. Yet neutral electrolyzed water (NEW) is generated by electrolysis in the membrane-less electrolytic cell. It produces a solution that is close to neutral pH (6–8) and has also been shown to be effective at reducing or eliminating bacterial pathogens [96]. Due to its different mechanisms of action, EOW has a wide range of activity. Electrolyzed water has been evaluated for several food safety industrial applications, including use as a fresh produce wash [97,98], cleaning and decontamination of dairy facilities [99], plant production [100], pig and poultry production and food facilities [101]. Cold fogging with electrolyzed water has been effective in decontamination of rooms mainly in health care environments [102]. A treatment with electrolyzed water applied by fogging was able to reduce MRSA and *Acinetobacter baumannii* [103]. In another study using an environmental-controlled chamber, Chuang et al. [104] sprayed a membrane-less electrolyzed water (MLEW) to reduce two airborne bacteria. They found prominent results to inactivate airborne bacteria using MLEW solution. Moreover, in an experimental hen house, the same authors reported the application of a fogging system releasing MLEW for bioaerosols decontamination [105]. In this study, 70% of the bacterial and fungal aerosols were reduced using a 10 min treatment of MLEW fogging.

The application of these treatments has been reviewed as a single treatment but a combination of several ones could improve their efficacy. An appropriate combination of techniques known as a hurdle concept has been effectively applied to enhance the microbial safety of food products and facilities.

## 4. Challenges and Future Considerations

Considerable evidence exists that environmental contamination with airborne microorganisms poses a risk for air–food transmission of these organisms. There are a number of promising treatments for food cold storage decontamination. The majority of these have low toxicity to humans and/or do not produce harmful by-products. In most instances, they are already in commercial and industrial use in the food industry or other sectors. Various companies are involved in design and development of novel decontamination techniques for food production facilities, namely, air ionization, ozone, ClO_2_, H_2_O_2_, with clear advantages over conventional techniques. These approaches will, however, require optimization for specific food application as well challenges regarding regulatory/legislative approval. Although current room decontamination methods might aid in controlling air contamination, there is still a need to promote the development of new practices or technologies in order to improve the whole room decontamination. The upsurge of microbial resistance to antibiotics and pesticides has increased the attention on novel technologies as alternative antimicrobial treatments. Numerous in vitro studies simulating indoor conditions have been performed involving airborne microorganism’s inactivation and new technologies approaches.

In some cases, scale up is an additional challenge for emerging technologies to be used in food industries. Research has to focus on different practical aspects of these techniques, including their impact on product quality and the economic costs.

There are a few studies in the literature assessing the efficacy of new methods for air decontamination; therefore, it is reasonable to continue research on their application. In addition, it will be useful to undertake studies on the cost effectiveness of the different technologies in order to facilitate their selection as a possible room decontamination technique. Furthermore, if new studies in this field continue to demonstrate good and consistent results, the introduction of these technologies should be considered for cold room decontamination in food facilities. Additionally, from the point of view of a food stakeholder, depending on the type and stage of food production operation, control of the aerial microbial is very challenging. However, it can be managed by continual air monitoring for microbial contamination together with an appropriate planning of HVAC (Heating, ventilation, and air conditioning), doorways, limitation of people movement inside rooms, supported by an appropriate air decontamination technology.

## Figures and Tables

**Table 1:** Current technologies for decontamination of air in cold storage room in food processing environment.

Application	Treatment Conditions	Target Microorganisms and Initial Load	Results	References
Fruit and vegetables cold storage rooms	Pulsed ultraviolet light (Xtend^®^ DeContam™ Alfa-01)—66 and 1000 s treatment Hydrogen peroxide by ultrasound fogging—1 h treatment	Total aerobic bacteria Moulds Initial values not reported	PUV significantly reduced the microbial load in the air of the storage room 66 s yielded similar results as 1000 s of treatment Stabilized H_2_O_2_ reduced microbial counts in the air on the day of treatment but an increase in air counts was observed after 24 h	[31]
Meat chill cooler	Germicidal air cleaning console unit (combination of filtration, electrostatic polarization and UV light) Several treatment times were used in combination with console unit	Total aerobic and facultative anaerobic bacteria Mould spores Initial values not reported	Reduction in 1 to 1.5 logs in airborne bacteria and moulds	[32]
Strawberry cold storage room	Chlorine dioxide Sodium hypochlorite Hydrogen peroxide Citric acid Ethanol By ultrasonic fogging—30 min of the fogging period and fog remained during 60 min treatment	Total aerobic bacteria—127.3 cfu Moulds—56.3 cfu	Reduced mould contamination to low levels compared to the untreated control	[33]
Pome fruit cold storage room	StorOx^®^ (mixture of hydrogen peroxide and peroxyacetic acid) by cold fogging	Moulds—200 cfu	The microorganism populations in the air were significantly reduced by all fogging applications	[34]
Fig cold storage room	Chlorine dioxide By ultrasonic fogging—30 min of the fogging period and fog remained during 60 min treatment	Total aerobic bacteria—425.0 cfu Moulds—762.4 cfu	The microorganism population was significantly reduced by fogging at 500 and 1000 µL/L Fogging at 1000 µL/L reduced fungal populations by more than 3.0 log units as compared to the control	[35]
Cheese storage room ^a^	Gaseous ozone	Moulds	88% reduction in mould spore counts	[36]
Cheese ripening room ^a^	Gaseous ozone	Moulds	Up to 99% decrease in viable counts of airborne moulds	[37]
Cheese ripening room	Gaseous ozone 8 g/h 6:00 p.m. to 6:00 a.m. for 5 weeks 4 g/h 6:00 p.m. to 6:00 a.m. for 2 weeks 8 g/h 40 min of each hour for 12 weeks	Moulds—524, 497 and 176 MPN/m^3^ depending of the sample area	A 10-fold reduction in the viable airborne mould loud (to <50 MPN/m^3^) compared with the control	[38]
Cheese ripening room	Gaseous ozone 0.48 mg de O_3_/m^3^ for 70% of the total time	Moulds—3.60 log cfu	After 40 days, a significant decrease in fungal viable counts, about 1.5 log	[39]
Cheese ripening room	Gaseous ozone 6 O_3_ generators of 30 mg/h for 60 days	Yeasts and moulds—10 cfu/plate	After 60 days of maturation, a decrease in viable yeasts and moulds counts were observed (3.7 cfu/plate)	[40]

^a^ No data available regarding treatment time and initial microbial load.

## References

[1] Parrett F., Crilly K. (2000). Microbiological air monitoring. Int. Food Hyg..

[2] Constantinidou H.A., Hirano S.S., Baker L.S., Upper C.D. (1990). Atmospheric dispersal of ice nucleation-active bacteria-the role of rain. Phytopathology.

[3] Lindemann J., Upper C.D. (1985). Aerial dispersal of epiphytic bacteria over bean-plants. Appl Env. Microbiol.

[4] Cevallos-Cevallos J.M., Gu G.Y., Danyluk M.D., Dufault N.S., van Bruggen A.H.C. (2012). *Salmonella* can reach tomato fruits on plants exposed to aerosols formed by rain. Int. J. Food Microbiol..

[5] Kwon Y.M., Woodward C.L., Pillai S.D., Pena J., Corrier D.E., Byrd J.A., Ricke S.C. (2000). Litter and aerosol sampling of chicken houses for rapid detection of *Salmonella* Typhimurium contamination using gene amplification. J. Ind. Microbiol. Biotechnol..

[6] Pearce R.A., Sheridan J.J., Bolton D.J. (2006). Distribution of airborne microorganisms in commercial pork slaughter processes. Int. J. Food Microbiol..

[7] Harbaugh E., Trampel D., Wesley I., Hoff S., Griffith R., Hurd H.S. (2006). Rapid aerosol transmission of *Salmonella* among turkeys in a simulated holding-shed environment. Poult. Sci..

[8] Proux K., Cariolet R., Fravalo P., Houdayer C., Keranflech A., Madec F. (2001). Contamination of pigs by nose-to-nose contact or airborne transmission of *Salmonella* Typhimurium. Vet. Res..

[9] Griffiths W.D., Decosemo G.A.L. (1994). The assessment of bioaerosols-A critical Review. J. Aerosol Sci..

[10] Roth Y., Chapnik J.S., Cole P. (2003). Feasibility of aerosol vaccination in humans. Ann. Otol. Rhinol. Laryngol..

[11] Sattar S.A., Ijaz M.K. (1987). Spread of viral-infections by aerosols. Crit. Rev. Environ. Contr..

[12] Ijaz M.K., Zargar B., Wright K.E., Rubino J.R., Sattar S.A. (2016). Generic aspects of the airborne spread of human pathogens indoors and emerging air decontamination technologies. Am. J. Infect. Control..

[13] McDermid A.S., Lever M.S. (1996). Survival of *Salmonella* Enteritidis PT4 and *Salmonella* Typhimurium Swindon in aerosols. Lett. Appl. Microbiol..

[14] Whyte P., Collins J.D., McGill K., Monahan C., O’Mahony H. (2001). Distribution and prevalence of airborne microorganisms in three commercial poultry processing plants. J. Food Prot..

[15] Spurlock A.T., Zottola E.A. (1991). The survival of *Listeria monocytogenes* in aerosols. J. Food Prot..

[16] Dutkiewicz J., Krysinska-Traczyk E., Skorska C., Sitkowska J., Prazmo Z., Golec M. (2001). Exposure to airborne microorganisms and endotoxin in herb processing plants. Ann. Agric. Environ. Med..

[17] Sawyer B., Elenbogen G., Rao K.C., Obrien P., Zenz D.R., Luehing C. (1993). Bacterial aerosol emission rates from municipal waste-water aeration tanks. Appl. Environ. Microbiol..

[18] Buckland F.E., Tyrell D.A.J. (1964). Experiments on the spread of colds. 1. Laboratory studies in dispersal of nasal secretions. J. Hyg..

[19] Burfoot D., Whyte R.T., Tinker D.B., Hall K., Allen V.M. (2007). A novel method for assessing the role of air in the microbiological contamination of poultry carcasses. Int. J. Food Microbiol..

[20] Burfoot D., Whyte R., Tinker D., Howell M., Hall K., Holah J., Smith D., White R., Baker D., McIntosh J. (2006). Importance of airborne contamination during dressing of beef and lamb carcasses. J. Food Protec..

[21] Sirami J. (1989). La contamination microbiologique de l’air dans le hall d’abbatage: Factures de variation et influence sur la carcasse. Viande Prod. Carne..

[22] Rahkio T.M., Korkeala H.J. (1997). Airborne bacteria and carcass contamination in slaughterhouses. J. Food Protec..

[23] Kang Y.J., Frank J.F. (1989). Biological aerosols: A review of airborne contamination and its measurement in dairy processing plants. J. Food Protec..

[24] Ye S.Y., Song X.L., Liang J.L., Zheng S.H., Lin Y. (2012). Disinfection of airborne spores of *Penicillium expansum* in cold storage using continuous direct current corona discharge. Biosyst. Eng..

[25] Okraszska-Lasica W., Bolton D.J., Sheridan J.J., McDowell D.A. (2012). Comparison of aerial counts at different sites in beef and sheep abattoirs and the relationship between aerial and beef carcass contamination. Food Microbiol..

[26] Ellerbroek L. (1997). Airborne microflora in poultry slaughtering establishments. Food Microbiol.

[27] Fries R., Graw C. (1999). Water and air in two poultry processing plant’s chilling facilities-a bacteriological survey. Br. Poult. Sci..

[28] Barkai-Golan R. (1966). Reinfestation of citrus fruits by pathogenic fungi in the packing house. Isr. J. Agric. Res..

[29] Marriott N.G., Gravani R.B. (2006). Principles of Food Sanitation.

[30] Kowalski W.J., Bahnfleth W.P., Striebig B.A., Whittam T.S. (2003). Demonstration of a hermetic airborne ozone disinfection system: Studies on *E. coli*. AIHA J..

[31] Saks Y., Ward G., Erdman S., Goldstein Y., Lichter A., Rodov V. (2006). Pulsed UV light for decontamination of cold storage facilities. Acta Hortic..

[32] Cundith C.J., Kerth C.R., Jones W.R., McCaskey T., Kuhlers D.L. (2002). Air-cleaning system effectiveness for control of airborne microbes in a meat-processing plant. J. Food Sci..

[33] Sholberg P.L. Bin and storage room sanitation. Proceedings of the Washington Tree Fruit Postharvest Conference.

[34] Vardar C., Ilhan K., Karabulut O.A. (2012). The application of various disinfectants by fogging for decreasing postharvest diseases of strawberry. Postharvest Biol. Technol..

[35] Karabulut O.A., Ilhan K., Arslan U., Vardar C. (2009). Evaluation of the use of chlorine dioxide by fogging for decreasing postharvest decay of Figure. Postharvest Biol. Technol..

[36] Gibson C.A., Elliot J.A., Beckett D.C. (1960). Ozone for controlling mold on cheddar cheese. Can. Dairy Ice Cream J..

[37] Shiler G.G., Eliseeva N.N., Chebotarev L.N. Use of ozone and ultra-violet radiation for the inactivation of mould spores. Proceedings of the 20th International Dairy Congress.

[38] Serra R., Abrunhosa L., Kozakiewicz Z., Venancio A., Lima N. (2003). Use of ozone to reduce molds in a cheese ripening room. J. Food Prot..

[39] Pinto A.T., Schmidt V., Raimundo S.A., Raihmer F. (2007). Moulds control by ozonization in ripening cheese room. Acta Sci. Vet..

[40] Lanita C.S., Silva S.B. (2008). Use of ozone in industrial cold rooms to control yeasts and moulds during parmesan cheese ripening. Braz. J. Food Technol..

[41] Malinowska A., Holah J. (2010). Whole Room Disinfection.

[42] Bintsis T., Litopoulou-Tzanetaki E., Robinson R.K. (2000). Existing and potential applications of ultraviolet light in the food industry-a critical review. J. Sci. Food Agric..

[43] Memarzadeh F., Olmsted R.N., Bartley J.M. (2010). Applications of ultraviolet germicidal irradiation disinfection in health care facilities: Effective adjunct, but not stand-alone technology. Am. J. Infect. Control..

[44] Rutala W.A., Weber D.J. (2011). Sterilization, high-level disinfection, and environmental cleaning. Infect. Dis. Clin. North. Am..

[45] Burfoot D. (1999). Clean air and a clean environment. Milk Ind. Inter..

[46] Holah J.T., Rogers S.J., Holder J., Hall K.E., Taylor J., Brown K.L. (1995). The Evaluation of Air Disinfection Systems.

[47] Shah B.P., Shah U.S., Siripurapu S.C.B. (1994). Ultraviolet irradiation and laminar air flow systems for clean air in dairy plants. Indian Dairym..

[48] Bodmer R. (1999). Hygiene durch UVC-Entkeimung [Hygiene with UV-C sterilization]. Fleischerei.

[49] Morgan R. (1989). UV ‘green’ light disinfection. Dairy Ind. Int..

[50] Hoehn R.C., Shorney-Darby H., Neemann J. (2010). Chlorine dioxide. White’s Handbook of Chlorination and Alternative Disinfectants, Black and Veatch Corporation.

[51] Tuladhar E., Terpstra P., Koopmans M., Duizer E. (2012). Virucidal efficacy of hydrogen peroxide vapour disinfection. J. Hosp. Infect..

[52] Morino H., Fukuda T., Miura T., Shibata T. (2011). Effect of low-concentration chlorine dioxide gas against bacteria and viruses on a glass surface in wet environments. Lett. Appl. Microbiol..

[53] Yeap J.W., Kaur S., Lou F.F., DiCaprio E., Morgan M., Linton R., Li J.R. (2016). Inactivation kinetics and mechanism of a human norovirus surrogate on stainless steel coupons via chlorine dioxide gas. Appl. Environ. Microbiol.

[54] Hedrick T.I. (1975). Engineering and science of aeromicrobiological contamination control in dairy plants. Chem. Ind..

[55] Burfoot D., Hall K., Brown K., Xu Y. (1999). Fogging for the disinfection of food processing factories and equipment. Trends Food Sci. Technol..

[56] McDonnell G., Russell A.D. (1999). Antiseptics and disinfectants: Activity, action, and resistance. Clin. Microbiol. Rev..

[57] Brul S., Coote P. (1999). Preservative agents in foods-Mode of action and microbial resistance mechanisms. Int. J. Food Microbiol..

[58] Smilanick J.L., Mansour M., Sorenson D. (2014). Performance of fogged disinfectants to inactivate conidia of *Penicillium digitatum* within citrus degreening rooms. Postharvest Biol. Technol..

[59] Johnston M.D., Lawson S., Otter J.A. (2005). Evaluation of hydrogen peroxide vapour as a method for the decontamination of surfaces contaminated with *Clostridium botulinum* spores. J. Microbiol. Methods.

[60] Tzanavaras P.D., Themelis D.G., Kika F.S. (2007). Review of analytical methods for the determination of chlorine dioxide. Cent. Eur. J. Chem..

[61] Vandekinderen I., Devlieghere F., Van Camp J., Kerkaert B., Cucu T., Ragaert P., De Bruyne J., De Meulenaer B. (2009). Effects of food composition on the inactivation of foodborne microorganisms by chlorine dioxide. Int. J. Food Microbiol..

[62] Huang J.L., Wang L., Ren N.Q., Ma F., Juli (1997). Disinfection effect of chlorine dioxide on bacteria in water. Water Res..

[63] Pillai K.C., Kwon T.O., Park B.B., Moon I.S. (2009). Studies on process parameters for chlorine dioxide production using IrO_2_ anode in an un-divided electrochemical cell. J. Hazard. Mat..

[64] Gómez-López V.M., Rajkovic A., Ragaert P., Smigic N., Devlieghere F. (2009). Chlorine dioxide for minimally processed produce preservation: A review. Trends Food Sci. Technol..

[65] Jeng D.K., Woodworth A.G. (1990). Chlorine dioxide gas sterilization under square-wave conditions. Appl. Environ. Microbiol..

[66] Wilson S.C., Wu C., Andriychuk L.A., Martin J.M., Brasel T.L., Jumper C.A., Straus D.C. (2005). Effect of chlorine dioxide gas on fungi and Mycotoxins associated with sick building syndrome. Appl. Environ. Microbiol..

[67] Daus A., Horev B., Dvir O., Ish-Shalom S., Lichter A. (2011). The efficacy of ultrasonic fumigation for disinfestation of storage facilities against postharvest pathogens. Postharvest Biol. Technol..

[68] Tapp C., Rice R.G., O’Donnell C., Tiwari B.K., Cullen P.J., Rice R.G. (2012). Generation and control of ozone. Ozone in Food Processing.

[69] Weavers L.K., Wickramanayake G.B., Block S.S. (2001). Disinfection and sterilization using ozone. Disinfection, Sterilization, and Preservation.

[70] Tiwari B.K., Rice R.G., O’Donnell C., Tiwari B.K., Cullen P.J., Rice R.G. (2012). Regulatory and legislative issues. Ozone in Food Processing.

[71] Cullen P.J., Tiwari B.K., O’Donnell C.P., Muthukumarappan K. (2009). Modelling approaches to ozone processing of liquid foods. Trends Food Sci. Technol..

[72] Khadre M.A., Yousef A.E., Kim J.G. (2001). Microbiological aspects of ozone applications in food: A review. J. Food Sci..

[73] Restaino L., Frampton E.W., Hemphill J.B., Palnikar P. (1995). Efficacy of ozonated water against various food-related microorganisms. Appl Environ. Microbiol..

[74] Kim J.G., Yousef A.E. (2000). Inactivation kinetics of foodborne spoilage and pathogenic bacteria by ozone. J. Food Sci..

[75] Taylor J., Chana D. (2000). The Evaluation of Ozone for Airborne and Surface Disinfection.

[76] Gabriel’yants’ M.A., Teplova L.N., Karpova T.I., Kozlova R.A., Makarova G.F. (1980). Storage of hard rennet cheeses in cold stores with ozonation of air. Kholodil’naya Tekhnika.

[77] Cullen P.J., Norton T., O’Donnell C., Tiwari B.K., Cullen P.J., Rice R.G. (2012). Ozone sanitisation in the food industry. Ozone in Food Processing.

[78] Fujishima A., Rao T.N., Tryk D.A. (2000). Titanium dioxide photocatalysis. J. Photochem. Photobiol. C Photochem. Rev..

[79] Horie Y., Taya M., Tone S. (1998). Evaluation of photocatalytic sterilization rates of *Escherichia coli* cells in titanium dioxide slurry irradiated with various light sources. J. Chem. Eng. Jpn..

[80] Matsunaga T., Tomoda R., Nakajima T., Wake H. (1985). Photoelectrochemical sterilization of microbial cells by semiconductor. FEMS Microbiol. Lett..

[81] Goswami D.Y., Trivedi D.M., Block S.S. (1997). Photocatalytic disinfection of indoor air. J. Sol. Energy Eng..

[82] Greist H.T., Hingorani S.K., Kelly K., Goswami D.Y. Using scanning electron microscopy to visualize photocatalytic mineralization of airborne microorganisms. Proceedings of the 9th International Conference on Indoor Air Quality and Climate Indoor Air 2002.

[83] Lin C.Y., Li C.S. (2003). Inactivation of microorganisms on the photocatalytic surfaces in air. Aerosol Sci. Technol..

[84] Wolfrum E.J., Huang J., Blake D.M., Maness P.C., Huang Z., Fiest J., Jacoby W.A. (2002). Photocatalytic oxidation of bacteria, bacterial and fungal spores, and model biofilm components to carbon dioxide on titanium dioxide-coated surfaces. Environ. Sci. Technol..

[85] Yu K.P., Lee G.W.M., Lin Z.Y., Huang C.P. (2008). Removal of bioaerosols by the combination of a photocatalytic filter and negative air ions. J. Aerosol Sci..

[86] Ye S.Y., Fan M.L., Song X.L., Luo S.C. (2010). Enhanced photocatalytic disinfection of *Penicillium expansum* in cold storage using a TiO_2_/ACF film. Int. J. Food Microbiol..

[87] Skowron K., Grudlewska K., Kwiecinska-Pirog J., Gryn G., Srutek M., Gospodarek-Komkowska E. (2018). Efficacy of radiant catalytic ionization to reduce bacterial populations in air and on different surfaces. Sci. Total Environ..

[88] Grinshpun S.A., Adhikari A., Honda T., Kim K.Y., Toivola M., Rao K.S.R., Reponen T. (2007). Control of aerosol contaminants in indoor air: Combining the particle concentration reduction with microbial inactivation. Environ. Sci. Technol..

[89] Ferro S., Coppellotti O., Roncucci G., Ben Amor T., Jori G. (2006). Photosensitized inactivation of *Acanthamoeba palestinensis* in the cystic stage. J. Appl. Microbiol..

[90] Friedberg J.S., Skema C., Baum E.D., Burdick J., Vinogradov S.A., Wilson D.F., Horan A.D., Nachamkin I. (2001). In vitro effects of photodynamic therapy on *Aspergillus Fumigatus*. J Antimicrob. Chemother..

[91] Lambrechts S.A.G., Aalders M.C.G., Van Marle J. (2005). Mechanistic study of the photodynamic inactivation of *Candida albicans* by a cationic porphyrin. Antimicrob. Agents Chemother..

[92] Zeina B., Greenman J., Purcell W.M., Das B. (2001). Killing of cutaneous microbial species by photodynamic therapy. Br. J. Derm..

[93] Maclean M., MacGregor S.J., Anderson J.G., Woolsey G. (2009). Inactivation of bacterial pathogens following exposure to light from a 405-nanometer light-emitting diode array. Appl. Environ. Microbiol..

[94] Landa-Solis C., Gonzalez-Espinosa D., Guzman-Soriano B., Snyder M., Reyes-Teran G., Torres K., Gutierrez A.A. (2005). Microcyn (TM): A novel super-oxidized water with neutral pH and disinfectant activity. J. Hosp. Infect..

[95] Vorobjeva N.V., Vorobjeva L.I., Khodjaev E.Y. (2004). The bactericidal effects of electrolyzed oxidizing water on bacterial strains involved in hospital infections. Artif. Organs.

[96] Rahman S.M.E., Khan I., Oh D.H. (2016). Electrolyzed water as a novel sanitizer in the food industry: Current trends and future perspectives. Compr. Rev. Food Sci. Food Saf..

[97] Abadias M., Usall J., Oliveira M., Alegre I., Viñas I. (2008). Efficacy of neutral electrolyzed water (NEW) for reducing microbial contamination on minimally-processed vegetables. Int. J. Food Microbiol..

[98] Pangloli P., Hung Y.C., Beuchat L.R., King C.H., Zha Z.H. (2009). Reduction of *Escherichia coli* O157:H7 on produce by use of electrolyzed water under simulated food service operation conditions. J. Food Prot..

[99] Wang X., Dev S., Demirci A., Graves R., Puri V. (2012). Electrolyzed Oxidizing Water for Cleaning-in-Place of Milking Systems on Dairy Farms.

[100] Hati S., Mandal S., Minz P.S., Vij S., Khetra Y., Singh B.P., Yadav D. (2012). Electrolyzed oxidized water (EOW): Non-thermal approach for decontamination of foodborne microorganisms in food industry. Food Nutr. Sci..

[101] Hung Y.C., Tilly P., Kim C. (2010). Efficacy of electrolyzed oxidizing (EO) water and chlorinated water for inactivation of *Escherichia coli* O157:H7 on strawberries and broccoli. J. Food Qual..

[102] Pintaric R., Matela J., Pintaric S. (2015). Suitability of electrolyzed oxidizing water for the disinfection of hard surfaces and equipment in radiology. J. Environ. Health Sci. Eng..

[103] Clark J., Barrett S.P., Rogers M., Stapleton R. (2006). Efficacy of super-oxidized water fogging in environmental decontamination. J. Hosp. Infect..

[104] Chuang C.Y., Yang S.H., Chang M.Y., Huang H.C., Luo C.H., Hung P.C., Fang W. (2013). Inactivation efficiency to *Bacillus subtilis* and *Escherichia coli* bacterial aerosols of spraying neutral electrolyzed water. J. Air Waste Manag. Assoc..

[105] Chuang C.Y., Fang W., Yang S.H., Chang M.Y., Hung P.C., Chang C.P. A study of membrane-less electrolyzed water fogging-spread for airborne bacteria and fungus decontamination in hen house. Proceedings of the 2011 International Conference on Agricultural and Biosystems Engineering.

